# Macrophage inducible C-type lectin (Mincle) recognizes glycosylated surface (S)-layer of the periodontal pathogen *Tannerella forsythia*

**DOI:** 10.1371/journal.pone.0173394

**Published:** 2017-03-06

**Authors:** Sreedevi Chinthamani, Rajendra P. Settem, Kiyonobu Honma, Jason G. Kay, Ashu Sharma

**Affiliations:** Dept. of Oral Biology, University at Buffalo, Buffalo, New York, United States of America; Medical University of South Carolina, UNITED STATES

## Abstract

The oral pathogen *Tannerella forsythia* is implicated in the development of periodontitis, a common inflammatory disease that leads to the destruction of the gum and tooth supporting tissues, often leading to tooth loss. *T*. *forsythia* is a unique Gram-negative organism endowed with an elaborate protein O-glycosylation system that allows the bacterium to express a glycosylated surface (S)-layer comprising two high molecular weight glycoproteins modified with O-linked oligosaccharides. The *T*. *forsythia* S-layer has been implicated in the modulation of cytokine responses of antigen presenting cells, such as macrophages, that play a significant role during inflammation associated with periodontitis. The macrophage-inducible C-type lectin receptor (Mincle) is an FcRγ-coupled pathogen recognition receptor that recognizes a wide variety of sugar containing ligands from fungal and bacterial pathogens. In this study, we aimed to determine if Mincle might be involved in the recognition of *T*. *forsythia* S-layer and modulation of cytokine response of macrophages against the bacterium. Binding studies using recombinant Mincle-Fc fusion protein indicated a specific Ca^2+^-dependent binding of Mincle to *T*. *forsythia* S-layer. Subsequent experiments with Mincle-expressing and Mincle-knockdown macrophages revealed a role for Mincle/S-layer interaction in the induction of both pro- and anti-inflammatory cytokine secretion in macrophages stimulated with *T*. *forsythia* as well as its S-layer. Together, these studies revealed Mincle as an important macrophage receptor involved in the modulation of cytokine responses of macrophages against *T*. *forsythia*, and thus may play a critical role in orchestrating the host immune response against the bacterium.

## Introduction

*Tannerella forsythia* is a Gram-negative oral anaerobe and a member of the so called ‘red complex’ bacterial consortium of the subgingival cavity strongly implicated in periodontitis [1, 2]. Periodontitis results from the damaging effects of the chronic inflammation against biofilm bacteria, leading to tooth loss. Inflammatory responses associated with chronic periodontitis also potentially contribute to the development of systemic diseases such as atherosclerosis, diabetes and arthritis [3, 4]. Several virulence factors that likely contribute to the pathogenicity of *T*. *forsythia* have been identified [5], and among them is the bacterium’s surface (S)-layer [6, 7]. S-layers are found as the outermost cell envelope in many bacteria and archaea, formed by the self-assembly of proteins into 2-D crystalline arrays [8]. S-layers generally function in the maintenance of bacterial integrity, display of bacterial components and interaction with the host non-immune and immune cells [9]. Strikingly, *T*. *forsythia* is the only Gram-negative bacterium to possess a glycosylated S-layer, which is formed by two extensively glycosylated proteins, TfsA and TfsB [10]. *T*. *forsythia* S-layer glycoproteins are modified with O-linked oligosaccharide branches containing a number of sugar residues: mannosaminuronic acid, fucose, xylose, digitose, galactose and rarely N-acetimidoyl (Am) modified pseudaminic acid (Pse) [11]. A potential role for *T*. *forsythia* S-layer in the modulation of immune response first became evident when it was observed that cytokine secretion by a human macrophage cell line was differentially induced in response to a S-layer deficient mutant of *T*. *forsythia* as compared to its parental strain [12]. Subsequently, we demonstrated that a terminal trisaccharide motif on S-layer proteins could play a role in blocking the recognition and processing of *T*. *forsythia* by dendritic cells [13].

Macrophages play a major role during inflammation associated with periodontitis [14]. Macrophages in periodontal lesions sites can drive bone resorption by producing osteoclastogenic favoring proinflammatory cytokines such as IL-1, IL-12, IL-6 and TNF-α [14]. The contribution of macrophages in periodontal bone loss is further supported by studies demonstrating that macrophage depletion significantly reduces alveolar bone resorption induced by *Porphyromonas gingivalis* infection [15]. Macrophages sense pathogen-associated molecular patterns (PAMPS) via germ line encoded pattern recognition receptors (PRRs) [16, 17]. Most studies have focused on the roles of toll-like receptors (TLRs) in pathogen sensing by macrophages during periodontal infections [18–20]. However, the contribution of a diverse class of PRRs, the C-type lectin receptors (CLRs) [21–23] that recognize microbial carbohydrate ligands has not been extensively explored in periodontitis. This is partly because glycosylation as a modification in periodontal pathogen molecules has not been fully appreciated, and is only beginning to be investigated with the availability of modern glycan analyses techniques. Macrophage-inducible C-type lectin (Mincle), also termed Clec4E and Clecsf9, is a key macrophage surface-expressed PRR with an extracellular carbohydrate recognition domain (CRD) [24]. Mincle recognizes diverse sugar-containing ligands including trehalose dimycolate glycolipid present of mycobacteria, mannose- or glucose-containing glycoconjugates of fungal pathogens [25], mannose-, or fucose- containing neoglycoconjugates, and Lewis antigen of *Helicobacter pylori* LPS [26]. Given that fucosylated sugar branches are linked to TfsA and TfsB proteins in *T*. *forsythia* S-layer [11], we investigated the possible involvement of Mincle as a receptor for the S-layer.

In this report through direct ligand binding assays we show that Mincle specifically binds S-layer glycoproteins leading to both pro- and anti-inflammatory cytokine secretion in macrophages. In addition, we show that Mincle signaling does not mediate bacterial uptake in macrophages. Together, our studies reveal Mincle as an important receptor for macrophage sensing of *T*. *forsythia* and modulation of immune responses to the bacterium.

## Materials and methods

### *T*. *forsythia* culture and S-layer purification

*T*. *forsythia* ATCC 43037 was grown anaerobically at 37°C in brain heart infusion (BHI) broth containing 5 μg/ml hemin, 0.5 μg/ml menadione, 0.001% N-acetylmuramic acid, 0.1% L-cysteine and 5% fetal bovine serum as described previously [27]. An S-layer-deficient *T*. *forsythia* mutant (*TfΔtfsAB*), generated by insertional inactivation of the S-layer genes in the ATCC 43037 [7], was obtained as a gift from Dr. Keiji Nagano, Aichi-Gakuin University, Japan. Intact native S-layer from *T*. *forsythia* was purified by cesium chloride density gradient ultracentrifugation according to a previously described protocol [6]. Briefly, *T*. *forsythia* cells were suspended in Tris-HCl pH 7.4 containing 2% sodium deoxycholate and stirred for 3 h at 4°C. The bacterial suspension was then centrifuged at 8000x*g* for 10 min at 4°C, supernatant collected and ultracentrifuged at 100,000x*g* for 1 h at 4°C. The pellet was resuspended in 50% CsCl in Tris-HCl (pH 7.4) and subjected to gradient centrifugation at 100,000x*g* for 18 h, revealing two major bands. The lower (heavier) band containing the S-layer was removed from the tube using an insulin syringe. The purified S-layer was then washed twice in Tris-HCl (pH 7.4), analyzed by SDS-PAGE to confirm quality (S1A Fig) and stored at −20°C until required.

### Macrophage differentiation and stimulation

The human monocytic cell line THP-1 was obtained from ATCC and maintained at 37°C in 5% CO_2_ in complete RPMI media comprising RPMI 1640 (Life Technologies, Grand Island, NY) with 10% FBS (Atlanta Biotech), 2 mM L-glutamine and 1% penicillin-streptomycin-neomycin solution (Life Technologies, Grand Island, NY). THP-1 cells were differentiated using 100 nM phorbol 12-myristate 13-acetate (PMA, Sigma-Aldrich) for 48 hours. After differentiation, PMA containing media was replaced with fresh complete RPMI and the cells were incubated for an additional 24 h prior to experiments.

### Mincle binding assay

Purified *T*. *forsythia* S-layer suspended in TBS (10 mM Tris-Cl, 0.1 M NaCl, pH 7.4) was coated onto 96-well plates (Maxisorb, Thermo Fisher Scientific) overnight at 4°C in the range of 0.325 to 25 μg/well. The coated wells were washed twice with TBS and blocked for 1 h at 37°C with Synblock blocking solution (AbD Serotec). Soluble Mincle-Fc (Antibodies-online Inc, Georgia) was added to the wells (100 μl of 1 μg/mL) in binding buffer (TBS, 5 mM CaCl_2_) or EDTA buffer (TBS, 10 mM EDTA) for 2 h at room temperature. Binding of Mincle to plate-bound trehalose-6, 6’-dibehenate (TDB, 5 μg/mL) was used as positive control. To confirm specificity, in some experiments human macrophage mannose receptor (MMR)-Fc (R&D Systems, MN, USA) construct and mycobacterium mannose-capped lipoarabinomannan (ManLAM; 5 μg/mL)(BEI Resources, VA, USA) were used as controls. For competitive inhibition assays Mincle-Fc was incubated for 10 min at room temperature with S-layer prior to incubation with plate bound TDB. Unbound Mincle-Fc (or MMR-Fc) was washed in each case with TBS and binding was determined by incubation with HRP-coupled goat anti-human IgG-Fc (Bethyl Laboratories, Texas) and chromogenic substrate TMB (KPL laboratories). Reaction was stopped with 0.1N H_2_SO_4_ and absorbance was read at 450 nm.

### siRNA knockdown of Mincle

Gene knockdown was carried out using Trilencer-27 siRNA pool against Mincle or a scrambled siRNA control (OriGene Technologies, MD). Briefly, prior to transfection differentiated THP-1 cells (2×10^5^) seeded in 24-well plates were incubated in serum free RPMI 1640 medium for 24 h. The cells were then transfected with Mincle or control siRNA using the Viromer Green transfection reagent as per the manufacturers recommendation (Lipocalyx, Halle, Germany). Five hours post transfection, the medium was replaced with fresh complete RPMI, and cells were maintained for another 72h.

### Quantitative real-time RT-PCR (qRT-PCR) and flow cytometry to assess Mincle expression

RNA from differentiated THP-1 cells were extracted with the RNeasy Mini kit (Qiagen), followed by treatment with DNase I (Qiagen) to remove residual DNA. The quality and integrity of purified RNA was routinely judged by denaturing agarose gel electrophoresis; presence of intact 28S and 18S rRNA bands indicated intact total RNA. All procedures were performed according to the manufacturer’s protocol. cDNA was then generated with the iScript cDNA Synthesis kit (Bio-Rad) in a 20 μl reaction volume with 500 ng of total RNA. The cDNA product was then diluted 10-fold and I μl of diluted sample was taken for each qPCR reaction, which was performed using iQ SYBR Green SuperMix (Bio-Rad) on the MyiQ cycler (Biorad). As a control to test for DNA contamination, total RNA without reverse transcriptase reaction was used in qPCR reaction with housekeeping control gene primers. Sequences of primer sets for the hypoxanthine-guanine phosphoribosyltransferase (HPRT; as housekeeping control) and Mincle mRNA expression were obtained from PrimerBank database [28]. qRT-PCR conditions included an initial 95°C incubation for 3 min followed by 40 cycle of 95°C for 30 sec, 60°C for 15 sec and 72°C for 15 sec. Mean fold changes were analyzed by 2^-ΔΔCT^ method [29]. To determine surface expression of Mincle, siRNA-treated (control or Mincle siRNA) differentiated THP-1 cells were detached with cell dissociation buffer (Gibco, Grand Island, NY) and incubated with 5 μg/mL mouse anti-mincle monoclonal antibody or isotype matched control (IgG2b) for 30 min at 4°C followed by incubation with FITC-labeled rat anti-mouse for 15 min at 4°C. Cells were washed with ice cold PBS containing 2% BSA and fluorescence was read by Fortessa (BD Biosciences, NJ) flow cytometer and results were analyzed by FCS software (De Novo Software, CA).

### Enzyme-linked immunosorbent assay

The concentration of TNF-α and IL-10 in cell culture supernatant were determined by ELISA kits according to the manufacturer’s instruction (eBiosciences).

### Determination of bacterial phagocytosis by a double-labeling technique

The contribution of Mincle to phagocytosis of *T*. *forsythia* cells was evaluated by comparing phagocytosis activity in control and Mincle-silenced THP-1 derived macrophages. Briefly, Mincle-silenced and control THP-1 derived macrophages seeded in 4-well cell culture chambers (Sigma Aldrich, MO), were incubated with bacteria at an MOI of 10. After incubation for 30 min at 37°C under 5% CO_2_, media was removed from the wells and cells were washed twice with cold PBS and incubated with rabbit anti-*T*. *forsythia* antiserum diluted 1:500 in PBS-0.5% BSA for 15 min at 4°C as described previously [30]. This was followed by washing wells with cold PBS containing 0.5% BSA and then incubation with goat anti-rabbit Ig coupled to Alexa Fluor 488 for 15 min at 4°C. Next, wells were washed twice with cold PBS/0.5% BSA and cells were fixed in 4% paraformaldehyde solution (Electron Microscopy Sciences, Washington, PA) for 30 min at room temperature. Following fixation, cells were washed one time with PBS and then permeabilized with 0.1% Triton X-100 solution for 5 min. Permeabilized cells were again incubated with rabbit anti-*T*. *forsythia* antiserum followed by Alexa Fluor-568 coupled goat anti-rabbit Ig to visualize internalized bacteria and DAPI nuclear stain for 30 min. Chambers slides after mounting were examined by Nicon Eclipse TE2000-u microscope and images were analyzed with SPOT imaging software (Diagnostics Imaging Inc, MI). The phagocytic index was calculated as: (total number of engulfed *T*. *forsythia* cells/total number of counted macrophages) x (number of macrophages with engulfed cells/total number of counted macrophages) x 100.

### Statistical analysis

Statistical differences were analyzed by ANOVA, and paired comparisons were performed by Tukey’s *post hoc* test. Statistical analyses were performed with the Prism Software (Graph Pad, San Diego, CA). Data were expressed as mean ± SD and differences were considered to be statistically significant at *P* < 0.05.

## Results

### *T*. *forsythia* and its S-layer bind to Mincle

**Fig 1:**
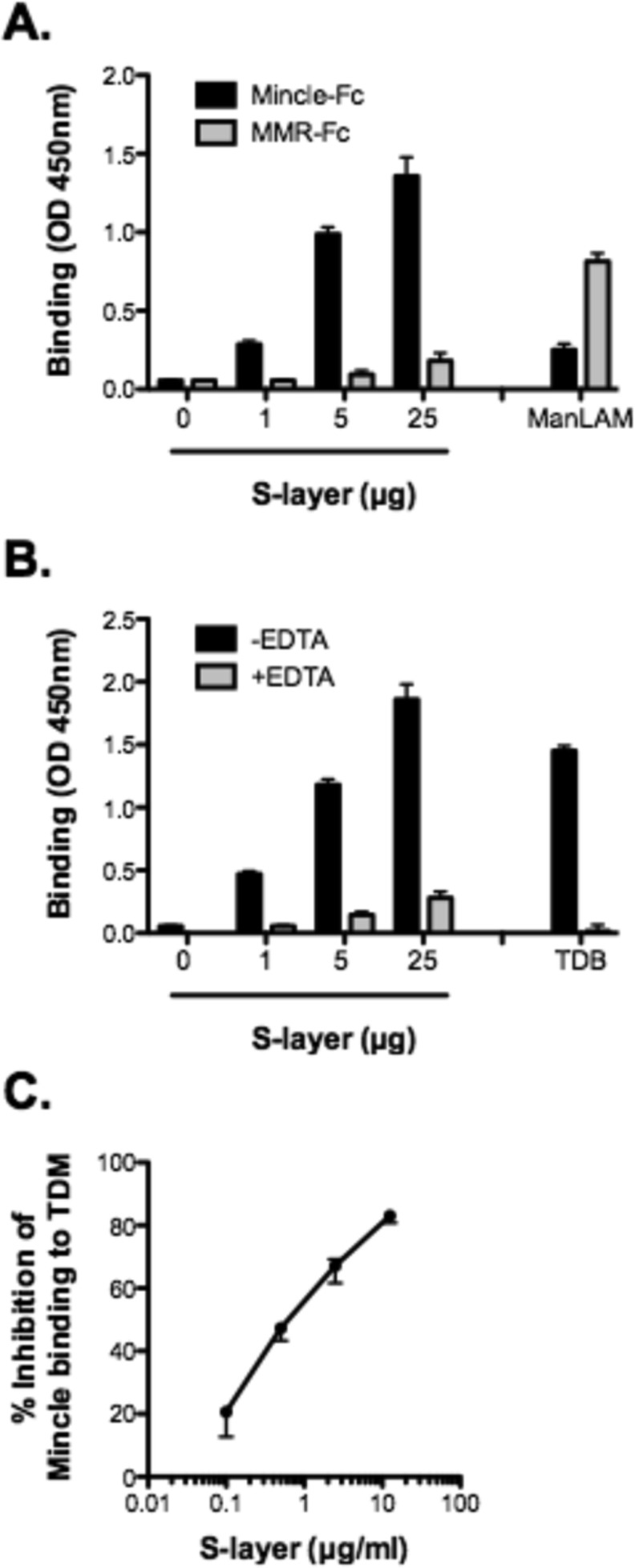
*T*. *forsythia* S-layer binds Mincle. (A) Binding of S-layer to Mincle. *T*. *forsythia* S-layer immobilized in microtiter plate wells was incubated with Mincle-Fc fusion protein or MMR-Fc in lectin binding buffer (TBS, 5 mM CaCl_2_). As control, ManLAM was immobilized in wells. After washing wells with buffer, binding was detected with HRP-coupled goat anti-human IgG-Fc and chromogenic substrate TMB. Color was read by measuring absorbance at 450 nm. As shown, S-layer bound Mincle in a dose dependent manner and MMR as control bound ManLAM but not S-layer. (B) Mincle binding to *T*. *forsythia* S-layer is Ca^2+^-dependent. To analyze the Ca^2+^ dependency of Mincle binding to S-layer, Mincle-Fc was incubated with plate bound S-layer in Ca^2+^ (TBS, 5 mM CaCl_2_) or EDTA containing buffer (TBS, 10 mM EDTA). Plate bound TDB was used a control ligand or Mincle binding. Binding was then detected as above. (C) S-layer dose dependently inhibited binding of Mincle to TDB. Plate bound TDB was incubated with Mincle-Fc in lectin binding buffer with increasing concentrations of S-layer. Wells were washed with elution buffer and binding was measured as above. Each value represents the mean (± SD) of 3 values measured in one representative assay. Data are representative of three independent experiments with similar results; *, P < 0.05.

Given the S-layer glycoproteins of *T*. *forsythia* are decorated with fucosylated sugar branches [11] (S1B Fig), we set out to determine whether the S-layer specifically binds Mincle and mediates *T*. *forsythia*-Mincle interactions. To test this we performed an ELISA-based binding assay with Mincle-Fc protein as a soluble receptor and plate bound S-layer and heat-inactivated bacteria as ligands. The results showed that Mincle-Fc construct bound to S-layer coated wells in a dose dependent manner (Fig 1A). Human macrophage mannose receptor (MMR)-Fc construct used as control exhibited little to no binding to plate bound S-layer, while strongly binding mycobacterium mannose-capped lipoarabinomannan (ManLAM), a positive control for MMR (Fig 1A).

While Mincle binds sugar containing ligands in a Ca^2+^-dependent manner, its binding to a protein ligand, the SAP130 protein, is Ca^2+^ independent [31, 32]. We sought to determine whether Mincle binding to S-layer was Ca^2+^-dependent, and thus mediated via sugars. Our results showed that incubation of Mincle-Fc in the presence of the Ca^2+^ chelating agent EDTA significantly reduced the binding of Mincle-Fc to S-layer glycoproteins (Fig 1B). Next, to confirm the specificity of Mincle to S-layer binding, we performed an ELISA-based competition assay with S-layer as a competitive inhibitor of Mincle binding to trehalose-6,6-dibehenate (TDB), a water soluble synthetic analog of the Mincle ligand trehalose-6,6-dimycolate [31]. The data showed that S-layer inhibited in a dose-dependent manner the binding of Mincle-Fc to TDB (Fig 1C). Taken together, we concluded that Mincle recognizes *T*. *forsythia* via S-layer glycoproteins in a calcium-dependent manner.

### *T*. *forsythia* S-layer differentially induces TNF-α and IL-10 secretion in macrophages

**Fig 2:**
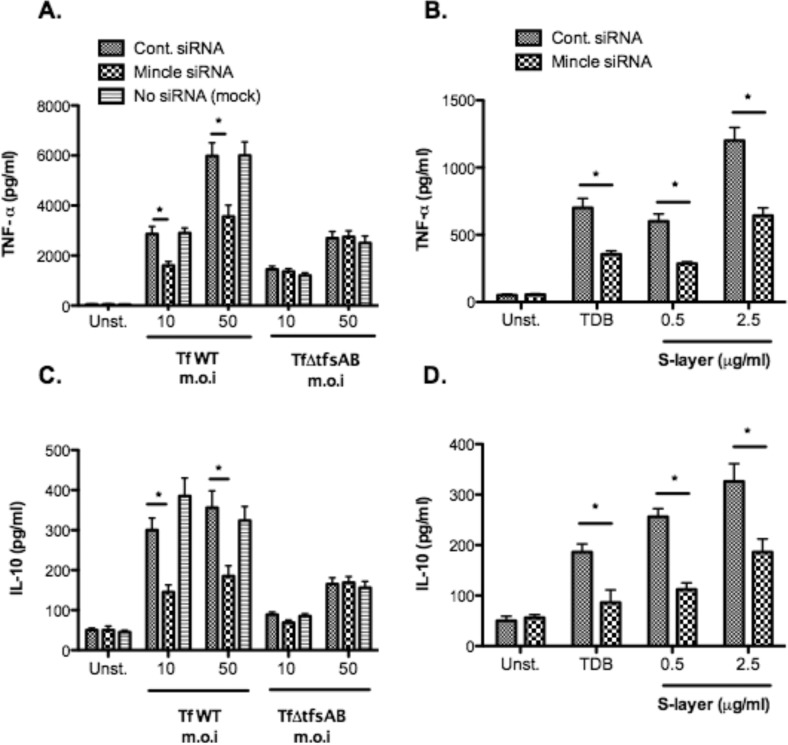
Mincle mediates pro- and anti-inflammatory cytokine secretion from bacteria- or S-layer-stimulated macrophages. Mincle +ve (control siRNA) and Mincle silenced (Mincle siRNA) macrophages were stimulated with *T*. *forsythia* at an MOI of 10 and 50, or S-layer (0.5μg/ml or 2.5μg/ml), for 12 h and cell culture supernatants were collected. The concentrations of TNF-α (A & B) and IL-10 (C & D) were determined by ELISA. Each value represents mean (± SD) of 3 values and the data are representative of three independent experiments with similar results.; *, P<0.05.

Next, we examined the functional significance of S-layer recognition by Mincle on macrophages. For this purpose, we determined the impact of Mincle expressed on THP-1 derived macrophages on cytokine expression in response to stimulation with S-layer. Mincle knockdown in THP-1 derived macrophage was achieved with Mincle specific siRNA. The data showed that in comparison to the control or mock (scrambled control) treated THP-1 cells, up to 90% reduction in Mincle mRNA expression with the Mincle specific siRNA was achieved (S2A Fig). The flow cytometry data further confirmed that Mincle siRNA treatment was effective in significantly reducing the surface expression of Mincle on THP-1 cells (S2B Fig). Normal and Mincle knockdown macrophages were then stimulated with whole bacteria, purified S-layer, or TDB (Mincle agonist) for 12 hours. In response to stimulation with purified S-layer or intact wild-type *T*. *forsythia* cells the secretion of TNF-α (Fig 2A & 2B) and IL-10 (Fig 2C & 2D) were significantly reduced in Mincle knockdown macrophages (Mincle siRNA) as compared to normal or mock (scrambled siRNA) treated THP-1 derived macrophages; and as expected and shown previously [33, 34], the secretion of TNF-α and IL-10 was significantly reduced in the Mincle knockdown cells in response to the Mincle ligand TDB (Fig 2B & 2D). Additionally, lower levels of cytokine secretion were observed from macrophages when challenged with the S-layer deficient *T*. *forsythia* mutant TfΔtfsAB than wild-type (Fig 2A & 2C) and furthermore, no further reductions were observed in cytokine release between Mincle knockdown and normal macrophages challenged with TfΔtfsAB (Fig 2A & 2C). These results indicated that the majority of the cytokine responses seen were due to Mincle recognizing the S-layer of *T*. *forsythia*, while the loss of S-layer likely causes exposure of other PAMPs involved in macrophage activation such as LPS and BspA allowing for significantly lower, but still higher than control, induction of both IL-10 and TNF-α (Fig 2A & 2C).

### Phagocytosis of *T*. *forsythia* in macrophages is Mincle independent

**Fig 3:**
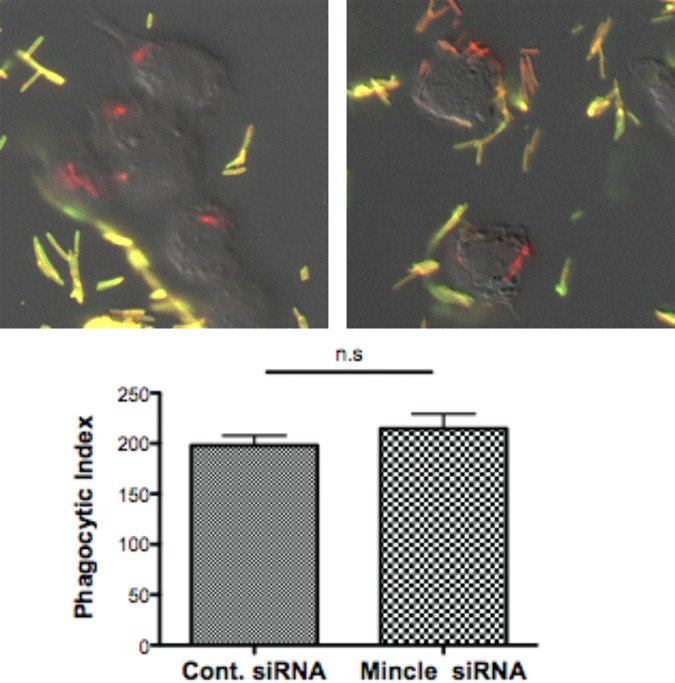
Phagocytosis of *T*. *forsythia* by macrophages is Mincle independent. *In vitro* phagocytosis of *T*. *forsythia* by scrambled siRNA (Mincle +ve) and Mincle silenced macrophages was performed and the phagocytic index was calculated. Top panels show a representative image of bacteria in macrophages observed by dual antibody labelling; internalized bacteria stained with Alexa-594 (red) and outside bacteria stained with Alexa-594 (red) and Alexa-488 (green), resulting in yellow on merged image. Bar graphs show percent phagocytic index for each condition. Each value represents mean (± SD) of 6 randomly selected fields in the slide with a minimum of 60 macrophages counted, and the data are representative of three independent experiments with similar results.; *, P<0.05.

Mincle has been shown to be important in the nonopsonic phagocytosis of *Klebsiella* by neutrophils [35]. On the other hand, only a limited role of Mincle in macrophages has been observed in the phagocytosis of *Streptococcus pneumoniae* [36]. To determine whether Mincle might be involved in the phagocytosis of *T*. *forsythia*, macrophages derived from scrambled siRNA or Mincle siRNA treated THP-1 cells were incubated with *T*. *forsythia* cells at an MOI of 10, and after incubation for 30 min at 37°C intracellular bacteria were quantified by dual antibody labeling immunofluorescence microscopy and phagocytic index calculated as described in Methods. The data showed (Fig 3) no significant difference in the phagocytosis of bacteria between control siRNA treated versus Mincle-siRNA treated macrophages, suggesting Mincle did not mediate phagocytosis of *T*. *forsythia* in macrophages. This also ruled out the possibility that differences in the cytokine responses seen was due to variable activation of intracellular PRRs occurring via phagocytosis.

## Discussion

Innate immune cells are constantly monitoring bacteria in the oral cavity to maintain oral health. In this regard, macrophage responses in particular are essential for the development of both innate and adaptive immunity to oral pathogens. Macrophages recognize pathogens and damaged host cell associated glycan motifs via the C-type lectin receptor family of PRRs. *T*. *forsythia* possesses an elaborate protein O-glycosylation system [37], which may allow the bacterium to display sugar structures for binding to lectin-like PRRs. In this regard, an OmpA-like protein with O-linked N-acetylhexosamine and hexose sugars was recently identified from *T*. *forsythia* [38]. This protein bound a variety of C-type lectin receptors including Selectins, Siglecs and DC-SIGN and mediated the association of the bacterium to oral epithelial cells via binding to P- and L-selectins [38]. Mincle is a macrophage expressed C-type lectin that recognizes sugar-containing ligands including trehalose glycolipids of mycobacteria [25], mannose- or glucose-containing glycoconjugates of fungal pathogens [25], LPS Lewis antigen of *H*. *pylori* [26], and mannose- or fucose-containing neoglycoproteins [39]. Given that S-layer glycoproteins of *T*. *forsythia* are decorated with fucosylated sugar branches [11], we set out to investigate Mincle as a putative receptor in the recognition of *T*. *forsythia*.

Our data shows that Mincle, in a Ca^2+^-dependent manner, bound purified S-layer glycoproteins as well as whole bacterial cells. Moreover, the binding of Mincle to its known ligand TDB was competitively inhibited with S-layer glycoproteins. Further, we found S-layer proteins were able to activate both pro- and anti-inflammatory cytokine secretion in THP-1 derived macrophages via Mincle, as siRNA knockdown of Mincle significantly suppressed TNF-α and IL-10 secretion in response to S-layer. As well, the TfΔtfsAB mutant lacking S-layer was significantly less able to induce cytokine expression by macrophages. Our results fit with reports demonstrating Mincle induction in macrophages by mycobacteria or TDM increases the production of both pro-inflammatory (TNF-α and nitric oxide) and anti-inflammatory (IL-10) responses [33]. Moreover, while *T*. *forsythia* S-layer has been shown previously to be involved in the activation of early proinflammatory cytokine response in macrophages [12], our study highlights the importance of S-layer sugars in macrophage activation via Mincle. Currently, mutants with sugar-specific deletions in the S-layer are unavailable to decipher the contribution of sugar moieties involved in macrophage interactions via Mincle.

Taken together, we demonstrate an important function of Mincle in modulating the innate immune responses to the important oral pathogen *T*. *forsythia* through induction of the anti-inflammatory cytokine IL-10. These findings may have broader relevance for *T*. *forsythia* infection and modulation of host immunity, as we have shown *T*. *forsythia* infection in mice causes suppression of Th1 but induction of Th2 biased responses [40]. IL-10 is an anti-inflammatory cytokine, which can act as a feedback regulator of Th1 and Th2 cell responses during infection, as well as suppresses IL-12 production in DCs and macrophages leading to a block in the development of Th1-type responses, compromising pathogen clearance [41–44]. Thus, Mincle-induced IL-10 could play a critical role in orchestrating T-cell immunity against *T*. *forsythia*. In addition, Mincle mediated signaling in TNF-α production may have direct implications on the pathogenesis of periodontitis. TNF-α, an inflammatory cytokine, is strongly implicated in the induction of tissue destructive responses occurring during periodontitis [45]. TNF-α triggers intracellular signaling pathways associated with apoptosis and inflammation [46, 47], TNF can stimulate osteoclastogenesis [48] and induce other inflammatory mediators including cytokines, chemokines, prostaglandins and adhesion molecules that together can cause connective tissue and alveolar bone loss during periodontitis [45]. Furthermore, we revealed that Mincle is not required for the phagocytic uptake of *T*. *forsythia* by macrophages. In summary, our studies reveal Mincle as an important receptor involved in sensing and modulation of the macrophage response against *T*. *forsythia*.

## Supporting information

S1 Fig *T. forsythia* S-layer glycoprotein characterization. (A) The *T*. *forsythia* S-layer glycoproteins were separated on sodium dodecyl sulfate-polyacrylamide gel electrophoresis (SDS-PAGE) (8% gels) and stained with glycostain (left) and probed with anti-S-layer antibody after western blotting (right). (B) O-glycan linked sugars on the S-layer glycoproteins of *T*. *forsythia*.
(PDF)

S2 Fig siRNA mediated knockdown of Mincle expression in THP-1 derived macrophages. (A) qRT-PCR results indicated reduced Mincle transcript levels 48 h after siRNA mediated knockdown (THP-1 siRNA). Scrambled siRNA was used as control. Data (means ± SD.) are representative of three independent experiments. Each value represents the mean (± SD) of 3 values measured in one representative assay; *, P < 0.05. (B) Reduction in the surface expression of Mincle as determined by flow cytometry. THP-1 derived macrophages after siRNA treatment were stained with a mouse monoclonal anti-Mincle antibody followed by FITC conjugated second antibody. Shown is a representative graph of three independent experiments with similar results.
(PDF)
