# Supplementary material for: Macrophage inducible C-type lectin (Mincle) recognizes glycosylated surface (S)-layer of the periodontal pathogen Tannerella forsythia
Source: PLoS One. 2017 Mar 6;12(3):e0173394. doi: 10.1371/journal.pone.0173394 (PMC5338828; doi:10.1371/journal.pone.0173394)
Supplement: S1 Fig — (A) The T. forsythia S-layer glycoproteins were separated on sodium dodecyl sulfate-polyacrylamide gel electrophoresis (SDS-PAGE) (8% gels) and stained with glycostain (left) and probed with anti-S-layer antibody after western blotting (right). (B) O-glycan linked sugars on the S-layer glycoproteins of T. forsythia. (PDF) [file pone.0173394.s001.pdf]

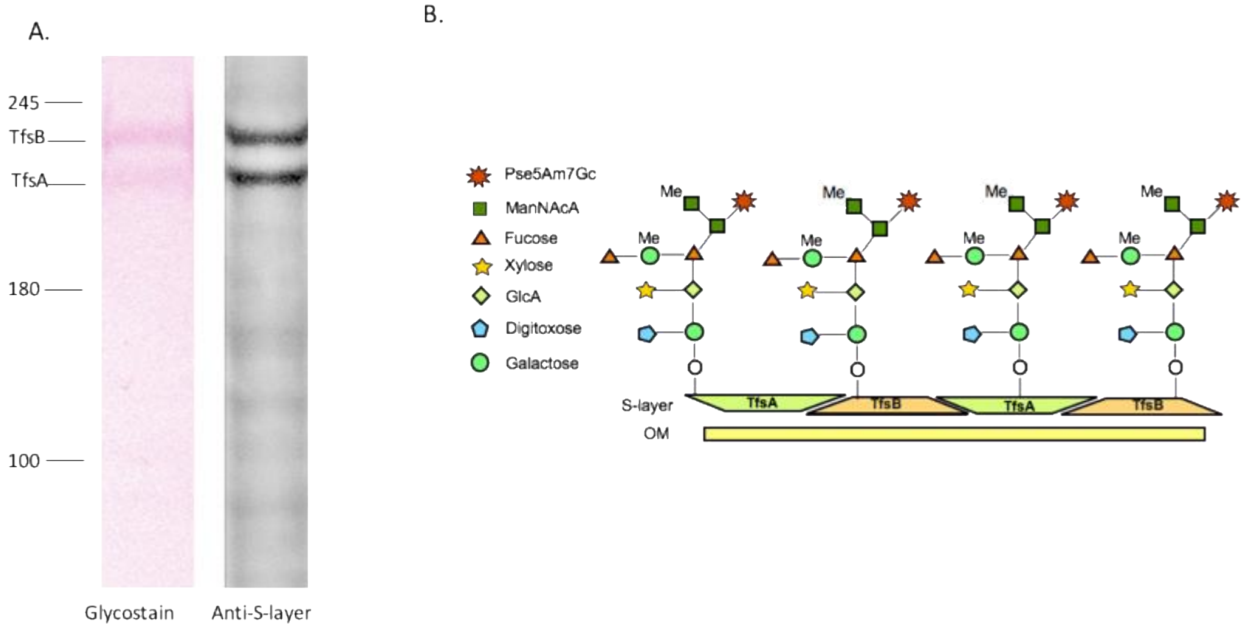

S1 Fig. *T. forsythia* S-layer glycoprotein characterization. (A) The *T. forsythia* S-layer glycoproteins separated on sodium dodecyl sulfate-polyacrylamide gel electrophoresis (SDS-PAGE) (8% gels) and stained with glycostain (left) and probed with anti-S-layer antibody [1] after western blotting (right). (B) O-glycan linked sugars on the S-layer glycoproteins of *T. forsythia* (determined in a previous study [2]).

1. Settem RP, Honma K, Nakajima T, Phansopa C, Roy S, Stafford GP, et al. A bacterial glycan core linked to surface (S)-layer proteins modulates host immunity through Th17 suppression. Mucosal immunology. 2013;6(2):415-26. doi: 10.1038/mi.2012.85. PubMed PMID: WOS:000317721400019.

2. Posch G, Pabst M, Brecker L, Altmann F, Messner P, Schaffer C. Characterization and scope of S-layer protein O-glycosylation in *Tannerella forsythia*. J Biol Chem. 2011;286:38714-24. Epub 2011/09/14. doi: 10.1074/jbc.M111.284893. PubMed PMID: 21911490.
