# Supplementary material for: Macrophage inducible C-type lectin (Mincle) recognizes glycosylated surface (S)-layer of the periodontal pathogen Tannerella forsythia
Source: PLoS One. 2017 Mar 6;12(3):e0173394. doi: 10.1371/journal.pone.0173394 (PMC5338828; doi:10.1371/journal.pone.0173394)
Supplement: S2 Fig — (A) qRT-PCR results indicated reduced Mincle transcript levels 48 h after siRNA mediated knockdown (THP-1 siRNA). Scrambled siRNA was used as control. Data (means ± SD.) are representative of three independent experiments. Each value represents the mean (± SD) of 3 values measured in one representative assay; *, P < 0.05. (B) Reduction in the surface expression of Mincle as determined by flow cytometry. THP-1 derived macrophages after siRNA treatment were stained with a mouse monoclonal anti-Mincle antibody followed by FITC conjugated second antibody. Shown is a representative graph of three independent experiments with similar results. (PDF) [file pone.0173394.s002.pdf]

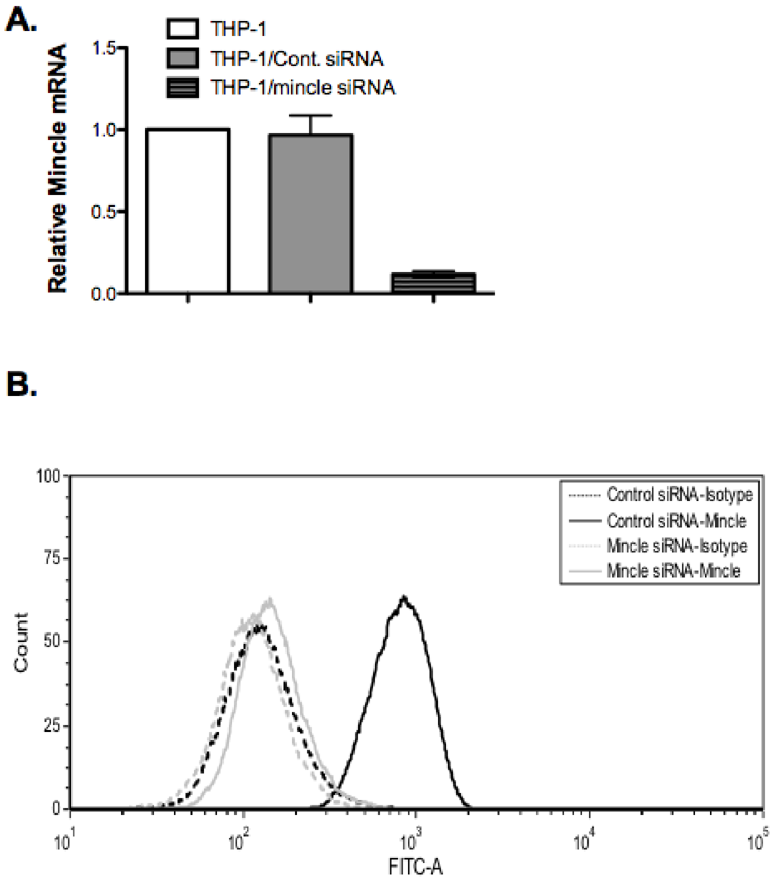


**S2 Fig. siRNA mediated knockdown of Mincle expression in THP-1 derived macrophages.** (A) qRT-PCR results indicated reduced Mincle transcript levels 48 h after siRNA mediated knockdown (THP-1 siRNA). Scrambled siRNA was used as control. Data (means ± s.d.) are representative of three independent experiments. Each value represents the mean (± SD) of 3 values measured in one representative assay; *, $P < 0.05$. (B) Reduction in the surface expression of Mincle as determined by flow cytometry. THP-1 derived macrophages after siRNA treatment were stained with a mouse monoclonal anti-Mincle antibody followed by FITC conjugated second antibody. Shown is a representative graph of three independent experiments with similar results.
